# Medicaid Coverage of Guidelines-Based Asthma Care Across 50 States, the District of Columbia, and Puerto Rico, 2021–2022

**DOI:** 10.5888/pcd20.230022

**Published:** 2023-09-07

**Authors:** Jacqueline Link, Hannah Green, Barbara Kaplan, Pamela Collins, Paige Welch, Carol Johnson

**Affiliations:** 1 American Lung Association, Washington, District of Columbia; 2 Centers for Disease Control and Prevention, National Center for Environmental Health, Atlanta, Georgia

## Abstract

**Introduction:**

Asthma affects more than 25 million Americans, including 4.2 million children. The burden of asthma disproportionately affects people enrolled in Medicaid, among other disparate groups. Improved availability and accessibility of guidelines-based treatments and services may ensure positive health outcomes for people with asthma. In this article, we provide an update to the American Lung Association’s Asthma Guidelines-Based Care Coverage Project (the Project) to determine the extent of asthma care coverage and associated barriers in Medicaid programs for all 50 states, the District of Columbia, and Puerto Rico, and examine improvements in coverage since 2017.

**Methods:**

Findings from the Project, representing coverage from 2016–2017, were first published in *Preventing Chronic Disease* in 2018. The Project was updated in 2021 to reflect the National Asthma Education and Prevention Program guidelines 2020 Expert Panel Report-3 updates, which were finalized in December 2020. It now tracks coverage for 8 areas of guidelines-based care and 7 barriers to care in Medicaid programs by reviewing publicly available plan documents and engaging with Medicaid programs to review and confirm findings.

**Results:**

Results from the Project, which reflect coverage in 2021–2022, show an increase in comprehensive coverage in Medicaid programs over the last 5 years. However, coverage remains inconsistent across programs, and barriers to accessing asthma care still exist.

**Conclusion:**

Although substantial improvement has been made to coverage, certain gaps and barriers to care must be addressed for patients to fully benefit from guidelines-based care to manage their asthma and improve health outcomes.

**Summary**

**What is already known on this topic?**

The American Lung Association’s Asthma Guidelines-Based Care Coverage Project (the Project) previously published its 2016–2017 asthma care coverage data in 2018 in *Preventing Chronic Disease*, finding that coverage in state Medicaid programs did not consistently adhere to National Asthma Education and Prevention Program guidelines.

**What is added by this report?**

This article updates this information with 2021–2022 asthma care coverage data that identifies both improvements in coverage and ongoing gaps in care.

**What are the implications for public health practice?**

We believe the Project will be of interest to public health practitioners, researchers, and policy makers aiming to improve asthma care by increasing equitable access to comprehensive asthma care coverage.

## Introduction

Treatment and services help control and manage asthma, which affects more than 25 million Americans, including 4.2 million children (1). Without proper treatment, asthma can be dangerous or fatal. Poorly managed asthma leads to nearly 2 million emergency department visits each year in the United States (2). Between 2008 and 2013, asthma accounted for $81.9 billion each year in economic costs in the US, with health care costs alone making up $50.3 billion of that estimate (3).

The burden of asthma highlights existing health disparities in the US. In 2020, Black Americans were 36% more likely to have asthma than White Americans (3). Asthma rates remain substantially higher among those with family incomes below the poverty threshold compared with those with incomes above it (1).

Additionally, asthma rates are higher among people enrolled in Medicaid compared with those with private insurance (12.4% vs 7.2%), highlighting the importance of access to guidelines-based asthma care in Medicaid programs (3). In 2018, Medicaid covered approximately 97 million people in the US at some point throughout the year, 43% of whom were children (4). Yet quality measures for asthma care show that many people with asthma enrolled in Medicaid struggle with asthma control. The percentage of people aged 5 to 64 years with an asthma medication ratio (ie, the ratio of controller medications to total asthma medications) with at least 75% medication compliance is 53.4% in commercial health maintenance organizations (HMOs) compared with only 39.1% in Medicaid HMOs (5). Ensuring that Medicaid programs adhere to guidelines-based care can improve asthma control and help to address health disparities related to asthma across the country.

The purpose of this analysis was to determine the extent of asthma care coverage and associated barriers in Medicaid programs in all 50 states, the District of Columbia (DC), and Puerto Rico; examine changes over the past 5 years, as national guidelines for asthma care have been updated; and identify opportunities for improvement.

## Methods

### The Asthma Guidelines-Based Care Coverage Project

To reduce the burden of asthma in the US, the Centers for Disease Control and Prevention (CDC) funds states and nongovernmental organizations to expand the reach of services through the National Asthma Control Program’s EXHALE strategies: **E**ducation on asthma self-management; e**X**tinguishing smoking and exposure to secondhand smoke; **H**ome visits for trigger reduction and asthma self-management education; **A**chievement of guidelines-based medical management; **L**inkages and coordination of care; and **E**nvironmental policies or best practices to reduce indoor and outdoor asthma triggers (6). The Asthma Guidelines-Based Care Coverage Project (the Project) supports strategy A, achievement of guidelines-based medical management, by raising awareness about coverage of asthma guidelines-based care among Medicaid and Children’s Health Insurance Program leaders, health care and public health professionals, and others in the asthma community.

The Project originally launched in 2015. Best practices for asthma care, in the context of this Project, were based on recommendations in the National Asthma Education and Prevention Program (NAEPP) Expert Panel Report 3 (7). The American Lung Association (Lung Association) convened a group of organizations to translate these standards into key components of guidelines-based asthma care that could be tracked in Medicaid programs and produced a benchmark document, Asthma Guidelines-Based Care Coverage Project: Benchmarks for Key Aspects of Optimal Coverage (8). Findings summarizing Medicaid coverage of guidelines-based asthma care in all 50 states, DC, and Puerto Rico from 2016–2017, the first-of-its-kind analysis of the extent to which Medicaid programs were covering guidelines-based asthma care, were published in *Preventing Chronic Disease* in 2018 (9).

### Updated guidelines

In December 2020, the NAEPP released the 2020 Focused Updates to the Asthma Management Guidelines: A Report from the National Asthma Education and Prevention Program Coordinating Committee Expert Panel Working Group (10). Topics included inhaled corticosteroids, long-acting muscarinic antagonists (LAMAs), immunotherapy, indoor allergen mitigation, fractional exhaled nitric oxide (FeNO) testing, and bronchial thermoplasty.

The Lung Association convened a second stakeholder group in 2020 to update the Project’s original benchmark document in response to the 2020 updates to the NAEPP guidelines, as well as lessons learned from the first 5 years of the Project. Key changes included the introduction of LAMAs as a category of controller medications, as well as the addition of other controller medications, modifications to clarify the definitions of home visiting and self-management education services, and the addition of a new category of coverage: lung function testing. In this new category, the Project tracks coverage of spirometry and FeNO testing. Several other changes were made to the benchmarks to clarify and simplify components and barriers based on the first 5 years of data collection for this Project (Table 1). 

**Table 1:** Categories of Care and Barriers to Accessing Care Defined, State Medicaid Program Coverage of Guidelines-Based Asthma Care^a^

Classification	Definition
**Category of care**	
Quick-relief medications	Fast-acting or quick-relief medications taken to provide immediate relief of bronchoconstriction and its accompanying acute symptoms.
Controller medications	Long-term control medications taken daily on a long-term basis to control persistent asthma.
Medical devices	Devices that assist with monitoring symptoms or properly administering asthma medication (includes nebulizers, peak flow meters, and valved holding chambers).
Allergy testing	Allergy tests for asthma triggers to identify and reduce exposure to allergens (skin or in vitro testing).
Allergy immunotherapy	Preventive treatment through incremental injected or ingested increases of the allergen to make the immune system less sensitive to the substance.
Lung function testing	Pulmonary function tests that check how well the lungs work. Spirometry measures the amount of air the lungs can hold. The test also measures how forcefully one can empty air from the lungs. Fractional exhaled nitric oxide (FeNO) testing can help to measure airway inflammation.
Home visits	An intervention that addresses the home environment, has at least 2 components, and includes assessment and education of either integrated pest control or at least 2 other asthma triggers.
Self-management education	People and families learn to use prescribed asthma-control medicines and equipment correctly, recognize symptoms of an asthma episode, and respond appropriately and mitigate asthma triggers.
**Barrier**	
Age limits	This barrier indicates that the treatment is only covered if an individual is a certain age, including if a treatment has additional barriers for people of certain ages. This barrier only applies provided it is more restrictive than NAEPP recommendations or FDA-approved guidelines.
Co-payments	This is a payment made for a specific service or treatment, even when it is covered by the insurance company (in this case Medicaid or Medicaid managed care plans).
Durable medical equipment (DME)	This means a device is covered only as DME, which could result in having to pay full price for the device at a retail pharmacy.
Eligibility criteria	This means a plan will only provide the treatment after a patient has experienced an incident(s), such as numerous visits to the emergency department.
Prior authorization	This barrier requires the provider to get approval from the insurance company (in this case Medicaid or Medicaid managed care plans) before the treatment will be covered (ie, paid for).
Quantity limits	This means that there is a limit on the number of treatments covered over a given period of time (ie, month or year).
Step therapy	This means a plan requires a patient to try and fail on a different treatment before the insurance company (in this case Medicaid or Medicaid managed care plans) will pay for the treatment that their provider prescribes.

Abbreviations: FDA, Food and Drug Administration; NAEPP, National Asthma Education and Prevention Program.

a Definitions of categories of asthma care and barriers to care as defined in the Asthma Guidelines-Based Care Coverage Project: Benchmarks for Key Aspects of Optimal Coverage (2020 Update) (11).

### Data collection

To determine NAEPP guidelines-based asthma care coverage and associated barriers in effect between July 1, 2021, and June 30, 2022, Lung Association staff examined Medicaid plan documents, formularies, preferred drug lists, member handbooks, and other publicly available related information for all 50 states, DC, and Puerto Rico. In many states, state Medicaid programs contract with managed care plans to provide coverage. An estimated 72% of all Medicaid enrollees receive coverage through managed care plans, with children and adults more likely than other Medicaid populations to be enrolled in a plan (12). In states with Medicaid managed care plans, Lung Association staff reviewed documentation for these plans as well, as coverage and barriers may differ from the state Medicaid plan’s coverage. Categories of coverage examined were quick-relief medications, controller medications, medical devices, allergy testing, allergy immunotherapy, lung function testing, home visits, and asthma self-management education (Table 1). Staff then sent data summaries to each Medicaid office to provide the opportunity to verify and confirm accuracy. Coverage values represent coverage across all standard Medicaid plans within each state (Table 2). To be marked as fully covered for a category (Y), every Medicaid plan in the state was confirmed to cover all treatments or services in that category. Varying coverage (V) in a category indicates that some but not all treatments or services within a category were covered, or that some but not all Medicaid plans in the state covered the treatments or services. A no (N) response was recorded if the state did not cover that category. If coverage of asthma treatments and services in a category could not be confirmed, that category was marked as not available (NA).

**Table 2:** State Medicaid Program Coverage of Guidelines-Based Asthma Care Categories 2021–2022^a^

State	Quick relief medication	Controller medication	Medical devices	Allergy testing	Allergy immunotherapy	Lung function testing	Home visits	Asthma self-management education
Alabama	Y	Y	V	Y	Y	Y	NA	Y
Alaska	Y	Y	Y	Y	Y	Y	N	Y
Arizona	V	V	V	V	V	V	NA	V
Arkansas	Y	V	Y	Y	Y	V	N	Y
California	Y	Y	V	Y	Y	V	V	V
Colorado	Y	Y	Y	Y	Y	Y	N	Y
Connecticut	Y	Y	V	Y	Y	Y	Y	Y
District of Columbia	Y	V	Y	Y	Y	Y	V	Y
Delaware	Y	V	V	Y	Y	Y	N	Y
Florida	Y	V	Y	Y	Y	V	NA	Y
Georgia	Y	Y	Y	Y	Y	Y	N	Y
Hawaii	V	V	V	V	V	V	NA	V
Idaho	Y	Y	Y	Y	Y	Y	N	Y
Illinois	Y	V	V	Y	Y	Y	N	V
Indiana	Y	Y	Y	Y	Y	Y	NA	Y
Iowa	Y	Y	Y	Y	Y	V	N	V
Kansas	Y	Y	Y	Y	Y	V	V	Y
Kentucky	Y	V	Y	Y	Y	V	NA	V
Louisiana	Y	V	Y	Y	Y	Y	N	V
Maine	Y	Y	Y	Y	Y	Y	N	Y
Maryland	V	V	V	V	V	V	NA	V
Massachusetts	Y	V	Y	Y	Y	Y	NA	Y
Michigan	Y	V	V	V	V	V	NA	V
Minnesota	Y	V	V	Y	Y	V	V	V
Mississippi	Y	Y	Y	Y	Y	Y	N	Y
Missouri	Y	Y	Y	Y	Y	V	Y	Y
Montana	Y	Y	Y	Y	Y	Y	N	Y
Nebraska	Y	Y	V	Y	Y	V	N	V
Nevada	V	V	Y	Y	Y	V	V	V
New Hampshire	Y	V	Y	Y	Y	Y	V	V
New Jersey	V	V	Y	Y	Y	V	N	V
New Mexico	V	V	Y	Y	Y	V	V	V
New York	V	V	V	Y	Y	V	NA	Y
North Carolina	Y	Y	V	V	V	V	NA	V
North Dakota	Y	Y	Y	Y	Y	Y	NA	Y
Ohio	Y	Y	V	Y	Y	Y	N	V
Oklahoma	Y	Y	Y	Y	Y	Y	N	Y
Oregon	V	V	V	V	V	V	NA	V
Pennsylvania	Y	Y	Y	Y	Y	V	V	Y
Puerto Rico	V	V	N	Y	Y	Y	N	NA
Rhode Island	Y	V	Y	Y	Y	Y	Y	Y
South Carolina	V	V	Y	Y	Y	V	V	Y
South Dakota	Y	Y	Y	Y	Y	Y	N	Y
Tennessee	Y	Y	V	V	V	V	N	V
Texas	Y	Y	Y	Y	V	V	V	V
Utah	V	V	Y	Y	Y	Y	N	Y
Vermont	Y	Y	Y	Y	Y	V	N	Y
Virginia	Y	Y	V	V	V	V	NA	V
Washington	Y	Y	Y	Y	Y	Y	N	V
West Virginia	Y	Y	Y	Y	Y	V	N	Y
Wisconsin	Y	Y	V	V	V	V	N	V
Wyoming	Y	Y	Y	Y	Y	Y	N	Y
**Asthma care coverage totals 2021–2022, no.**								
Yes	41	29	33	43	42	25	3	28
Varies	11	23	18	9	10	27	10	23
No/NA	0	0	1	0	0	0	39	1

a N indicates that none of the items in the category is covered in any Medicaid plan; NA indicates that data were not available for the state; V indicates that not all items in the category were covered in all Medicaid plans (some items in category may have varying coverage across plans or no coverage at all); and Y indicates that all items in the category of care are covered in all Medicaid plans.

If the Medicaid program covered the treatment or service in any capacity, barrier data were assessed. These barriers were age limits, co-payment, durable medical equipment, eligibility criteria, prior authorization, quantity limits, and step therapy (Table 1). Percentages were calculated using Excel (Microsoft Corporation) by dividing the number of programs with a specific barrier by the total number of programs with coverage or varying coverage of a specific category (Table 3). For example, only 13 programs offered any form of coverage for home visits. Of those 13, six indicated that eligibility criteria were a barrier to coverage, so the frequency of this barrier in the home visit category was 6 of 13, or 46.2%, meaning that eligibility criteria were a barrier to home visits for 46.2% of Medicaid programs that covered the service. In categories with multiple components, if a given barrier appeared in any of the components, that barrier was marked as appearing in that category of coverage (Table 3). For example, if omalizumab required prior authorization, the entire controller medication category would be marked as requiring prior authorization in the given program, even if the other controller medications in that category did not require it. Programs had to confirm that barriers were not present in all components of a category to be marked as not having a given barrier.

**Table 3:** Frequency of Barriers to Coverage of Guidelines-Based Asthma Care, by Category^a^

Barrier	Quick relief medication	Controller medication	Medical devices	Allergy testing	Allergy immunotherapy	Self-management education	Home visits	Lung function testing
Age limits	15.4	48.1	5.9	0	0	17.6	38.5	3.8
Co-payment	71.2	71.2	58.8	30.8	28.8	19.6	0	25.0
Durable medical equipment	NA	NA	94.1	NA	NA	NA	NA	NA
Eligibility criteria	NA	43.1	NA	NA	NA	5.9	46.2	0
Prior authorization	82.7	98.1	29.4	9.6	13.5	9.8	23.1	15.4
Quantity limits	78.8	86.5	88.2	28.8	32.7	25.5	38.5	23.1
Step therapy	73.1	88.5	NA	NA	NA	NA	NA	NA

Abbreviation: NA, not applicable.

a Frequency of barriers is calculated by dividing the confirmed number of states with the barrier by the total number of states that cover the given category of care. All values are percentages.

## Results

### Coverage

Most programs offered some form of coverage for 7 of the 8 components of guidelines-based care, a substantial improvement from 2016–2017 (Table 2) (Figure). Allergy testing was the most widely covered category of care (n = 43 Medicaid programs), followed closely by allergy immunotherapy (n = 42 Medicaid programs). Quick-relief medication also was widely covered, with 41 Medicaid programs offering coverage of both types of quick-relief medication in both forms (nebulized and inhaled). Home visits were the least-covered category of guidelines-based care, with only 3 Medicaid programs covering visits that assess the home environment, and 10 more Medicaid programs with coverage that varied by plan.

**Figure:**
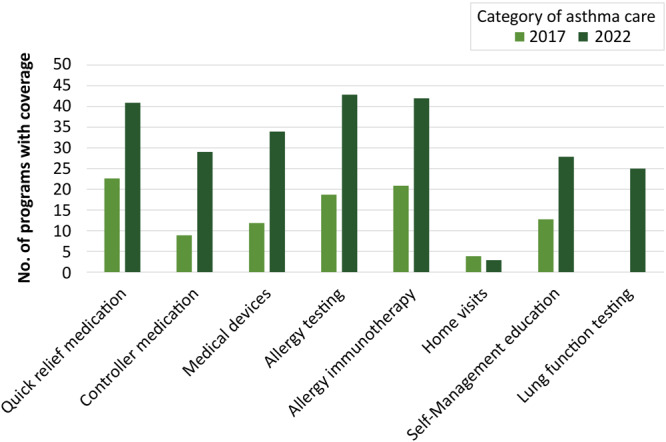
Coverage of guidelines-based asthma care categories in 2017 and 2022. Data were not collected for lung function testing in 2017.

**Table:** 

Category of asthma care	Quick relief medication	Controller medication	Medical devices	Allergy testing	Allergy immunotherapy	Home visits	Self-management education	Lung function testing
	Number of programs with coverage							
**Coverage in 2017**	23	9	12	19	21	4	13	—
**Coverage in 2022**	41	29	34	43	42	3	28	25

Given that many categories required several components of coverage to be considered fully covered, many programs were marked as having coverage that varied because they were missing 1 of those components. For example, for quick-relief medications, although all 50 states, DC, and Puerto Rico fully covered albuterol in nebulized and inhaled forms, only 41 of those Medicaid programs also covered both forms of levalbuterol. Coverage for lung function testing also varied. Thirty-five Medicaid programs fully covered spirometry, and 17 more had coverage that varied by plan. In comparison, only 25 Medicaid programs had full coverage of FeNO testing, 24 Medicaid programs had coverage that varied by plan, and 3 did not cover FeNO testing at all, though this may be because this testing was first recommended in the 2020 Focused Updates to the Asthma Management Guidelines (10). Programs should cover all components of a guidelines-based category to ensure the best health outcomes for people with asthma.

When compared with data collected on guidelines-based coverage in 2016–2017, there has been a substantial improvement in coverage across all categories of care except for home visits (Figure). The largest increase in coverage can be seen in allergy testing: in 2017 only 19 Medicaid programs fully covered this service, but in 2022, 43 Medicaid programs covered it fully. In every category of guidelines-based coverage except home visits and lung function testing, there was an increase of at least 15 new Medicaid programs with full coverage in the given category between 2017 and 2022. The only category where no improvement can be seen is asthma home visits.

### Barriers

Barriers to guidelines-based care in all categories were assessed where coverage could be confirmed. The most frequent barriers noted in the medications categories were prior authorization, quantity limits, and step therapy (Table 3). The most frequent barrier noted in the medical devices category was durable medical equipment, with 94% of Medicaid programs having this barrier to coverage for at least 1 device. For allergy testing, allergy immunotherapy, lung function testing, and asthma self-management education, the most frequent barriers to coverage were co-payments and quantity limits. Among the 13 Medicaid programs that had at least 1 plan that covered home visits, eligibility criteria were the most common barrier.

## Discussion

### Opportunities to further improve guidelines-based asthma care coverage and access

Improving access to guidelines-based asthma care can result in better patient outcomes, including reduced asthma emergencies and associated health care costs (13). The findings of this Project show that, although there has been substantial improvement in coverage of some care categories, large gaps in guidelines-based asthma care coverage continue among Medicaid programs.

Furthermore, barriers to care like those examined in this Project can worsen health outcomes for people with asthma. People can be delayed or denied access to proper care while waiting for prior authorizations and step therapies to be completed by Medicaid. Quantity limits can make it more difficult for people with asthma to access the medications they need, or to replace necessary medical equipment. This is especially true for children and youth who may need multiple inhalers to treat their asthma in home and school settings. Navigating the processes to remove barriers may be time-consuming and difficult to understand, especially for patients who are unfamiliar with the health care system. Removing barriers to coverage such as prior authorizations and quantity limits may improve health outcomes and equitable access to care for people with asthma.

Another opportunity to improve access to guidelines-based care for people with asthma and providers involves the transparency of coverage information. When completing data collection for this Project, many programs did not have coverage information readily accessible, including detailed preferred drug lists, prior authorization requirements, or service code lists. This lack of information can, in itself, be a barrier to care if people with asthma and providers are unable to confirm coverage or the correct path to coverage. In collecting data for this Project, the Lung Association also had difficulty reaching some Medicaid offices for verification of coverage. Transparency of coverage information is important to maintaining standards of care across Medicaid programs.

Medicaid programs and Medicaid managed care plans can improve access to asthma guidelines-based care by using data in this Project to identify gaps in their coverage and improve accessibility of care for Medicaid beneficiaries.

### Challenges to improving coverage

Although the motivations for improvements in coverage (or lack thereof) are outside the scope of this analysis, the cost of providing certain recommended treatments and services for asthma likely plays a role in the coverage decisions of state Medicaid programs and managed care plans. The short-term cost of improving coverage may deter some stakeholders from making changes, but improving accessibility and breadth of coverage can have financial benefits for Medicaid programs as well. Comprehensive coverage of pediatric asthma care is associated with lower costs for Medicaid programs providing coverage (14). Expanding asthma coverage to include home visits and other services has the potential to reduce costs, particularly from reduced pediatric emergency department visits (15).

Home visits were the least covered and least improved category of asthma care. There are plans that covered some supplies or provided some components of a home visit but did not meet the full criteria for a minor intensity home visit as defined in the Project’s benchmark document. Although the Project examines only barriers to coverage, not barriers to implementation, implementation barriers likely play a role in the lack of coverage uptake (16).

Other challenges that Medicaid programs and Medicaid managed care plans may also face in addressing gaps in coverage include gathering data necessary to track progress, securing sufficient reimbursement rates, and certifying asthma educators or other professionals needed to deliver guidelines-based care through Medicaid programs (17). Through this Project, the Lung Association also produces technical assistance resources to help stakeholders improve coverage of guidelines-based asthma care through Medicaid programs.

### Effects of COVID-19 pandemic

The COVID-19 pandemic continues to pose unprecedented challenges for people with lung disease, including asthma. Following the start of the pandemic, in-person asthma emergency department visits and hospitalizations significantly decreased (87%), while the use of telehealth visits (video and phone calls) increased among people with asthma (18). However, this temporary decrease in emergency department and inpatient visits was not due to an improvement in asthma control but to other pandemic-related factors.

At the policy level, the COVID-19 pandemic and the subsequent public health emergency had effects on the functioning of Medicaid programs providing asthma care coverage. Many programs increased the availability of telehealth as an alternative to in-person appointments (19). Some programs suspended existing barriers including co-payments, quantity limits, and prior authorizations because of the public health emergency (20). If these were temporary measures, programs that suspended these barriers were still considered to have these barriers during the data collection for the Project. While the official public health emergency has ended, programs still have the ability to render those changes permanent to the benefit of people with asthma.

### Limitations of data collection

Although the increase in guidelines-based coverage is a promising trend for people with asthma, the Project’s methods and benchmarks have changed in many ways since our 2016–2017 analysis. These changes are due to the 2020 updates to the NAEPP guidelines and lessons learned from the first 5 years of the Project. Lung function testing has been added as a category, and several changes have been made to the other categories of coverage. Two quick-relief medications (anticholinergics) have been removed from the quick-relief medication category, and both the nebulized and inhaled forms of albuterol and levalbuterol are now tracked. Two medications have been removed from the controller medications category, and 3 others were added. The allergy testing category no longer distinguishes between in vitro testing and skin testing. The definitions for coverage of home visits and self-management education have been refined to provide greater detail and clarity.

### Conclusion

Ultimately, improving coverage of guidelines-based asthma care through Medicaid programs will help people with asthma achieve better health outcomes and improve health equity. This analysis has demonstrated that coverage of guidelines-based asthma care through Medicaid has improved in the 5 years between updates, but work remains to be done. The Project will continue to focus on opportunities for collaboration and continued progress in improving health outcomes and health equity among people with asthma.
